# It takes a village: An empirical analysis of how husbands, mothers‐in‐law, health workers, and mothers influence breastfeeding practices in Uttar Pradesh, India

**DOI:** 10.1111/mcn.12892

**Published:** 2019-11-26

**Authors:** Melissa F. Young, Phuong Nguyen, Shivani Kachwaha, Lan Tran Mai, Sebanti Ghosh, Rajeev Agrawal, Jessica Escobar‐Alegria, Purnima Menon, Rasmi Avula

**Affiliations:** ^1^ Hubert Department of Global Health Emory University Atlanta GA USA; ^2^ Poverty, Health and Nutrition Division International Food Policy Research Institute (IFPRI) Washington DC USA; ^3^ FHI360 Washington DC USA

**Keywords:** breastfeeding initiation, exclusive breastfeeding, India, prelacteal feed, programme, Uttar Pradesh

## Abstract

Evidence on strategies to improve infant and young child feeding in India, a country that carries the world's largest burden of undernutrition, is limited. In the context of a programme evaluation in two districts in Uttar Pradesh, we sought to understand the multiple influences on breastfeeding practices and to model potential programme influence on improving breastfeeding. A cross‐sectional survey was conducted among 1,838 recently delivered women, 1,194 husbands, and 1,353 mothers/mothers‐in‐law. We used bivariate and multivariable logistic regression models to examine the association between key determinants (maternal, household, community, and health services) and breastfeeding outcomes [early initiation of breastfeeding (EIBF)], prelacteal feed, and exclusive breastfeeding (EBF). We used population attributable risk analysis to estimate potential improvement in breastfeeding practices. Breastfeeding practices were suboptimal: EIBF (26.3%), EBF (54%), and prelacteal feeding (33%). EIBF was positively associated with maternal knowledge, counselling during pregnancy/delivery, and vaginal delivery at a health facility. Prelacteal feeds were less likely to be given when mothers had higher knowledge, beliefs and self‐efficacy, delivered at health facility, and mothers/mothers‐in‐law had attended school. EBF was positively associated with maternal knowledge, beliefs and self‐efficacy, parity, and socio‐economic status. High maternal stress and domestic violence contributed to lower EBF. Under optimal programme implementation, we estimate EIBF can be improved by 25%, prelacteal feeding can be reduced by 25%, and EBF can be increased by 23%. A multifactorial approach, including maternal‐, health service‐, family‐, and community‐level interventions has the potential to lead to significant improvements in breastfeeding practices in Uttar Pradesh.

**Key messages**

- Focus is needed to improve breastfeeding practices in Uttar Pradesh as only a quarter of infants are fed within the first hour of birth, half of infants are exclusively breastfed in the first 6 months, and a third of infants receive a prelacteal feed.
- Programme investments to engage men and mothers‐in‐law, at the individual, household, community levels are required to address the complex and multifactorial factors that influence breastfeeding practices in this context.
- Continued promotion of facility delivery and breastfeeding counselling during pregnancy, delivery, and postpartum are recommended given the promising association with improved breastfeeding practices. Further health system efforts may be merited to help support breastfeeding among women who deliver via C‐section.
- Under optimal programme implementation and conditions, we estimate early initiation of breastfeeding can be improved by 25%, prelacteal feeding can be reduced by 25%, and exclusive breastfeeding can be increased by 23%.

## 1 INTRODUCTION

Breastfeeding is one of the most cost‐effective child survival interventions known; an estimated of 823,000 children, and 20,000 women's lives would be saved annually if breastfeeding practices were scaled up globally (Victora et al., 2016). Breastfeeding in the first 2 years of life sets the stage for a lifetime as it protects infants from morbidity, mortality, and may reduce obesity and diabetes later life (Victora et al., 2016). World Health Organization (WHO) recommends early initiation of breastfeeding (EIBF) within first hour and exclusive breastfeeding (EBF) for first 6 months with continued breastfeeding up to 2 years or beyond, with the addition of nutritionally and age appropriate complementary foods (WHO, 2003). Given the importance of breastfeeding, the global nutrition targets for 2025 adopted by the World Health Assembly call to increase EBF to at least 50% (International Food Policy Research Institute, 2016). Despite political and programme prioritisation, most countries are off course for meeting this target.

Globally, 42% of infants are breastfeed within the first hour, and 41% of infants are currently exclusively breastfeed for the first 6 months (UNICEF, 2018). In India, breastfeeding rates have improved in the last decade. In India, EIBF nearly doubled from 23% to 42%, and EBF increased from 46% to 55% between 2005–2006 and 2015–2016 [International Institute for Population Sciences (IIPS), 2007; IIPS, 2017a]. Despite progress, disparities across India remain and further progress is needed (Nguyen et al., 2018). In Uttar Pradesh, breastfeeding rates are much lower compared with the national average with EIBF at only 7% in National Family Health Survey (NFHS)‐3 (2005–2006; IIPS, 2017b) and increasing to 25% in NFHS 4 (2015–2016); however, in this period, EBF rates decreased from 51% to 42%, slipping below global targets (IIPS, 2017b). To address these challenges, India has developed comprehensive infant and young child feeding programmes and policies that are well aligned with global guidance (Avula, Oddo, Kadiyala, & Menon, 2017; Bhutta et al., 2013; India‐MoHFW, 2013; MoWCD, 2013; Vir et al., 2014). More recently, India's new nutrition strategy and programme efforts (Ministry of Women and Child Development, 2018; National Institution for Transforming India Aayog, 2017) place interventions to address infant and young child feeding at the heart of efforts to improve nutrition. However, a review of the policy environment in India identified key implementation barriers such as lack of clear operational guidance, insufficient use of monitoring data to inform programme activities, and training/supervision capacity gaps (Avula et al., 2017).

To improve breastfeeding practices, the recent Lancet breastfeeding series outlines the multifactorial and complex factors that are required to best support women and create an enabling environment at the societal level, within the health system, in the community/workplace, and within families (Rollins et al., 2016). Although globally, the determinants of breastfeeding are well established, there is a critical need for a contextualised understanding of the relative contribution of these factors in local contexts to help inform programme operations. Furthermore, although qualitative studies and reports from mothers have suggested a strong influence of mothers/mothers‐in‐law (MMILs) and husbands on breastfeeding practices (Bromberg Bar‐Yam & Darby, 1997; Negin, Coffman, Vizintin, & Raynes‐Greenow, 2016), few studies examined the role of MMILs and husbands in supporting women to breastfeed. To address these gaps in the literature, and to inform a specific programmatic context in Uttar Pradesh, India, this paper aims to examine the key maternal, household, community, and health service factors that influence breastfeeding practices in Uttar Pradesh.

## 2 METHODS

### 2.1 Data sources and study population

We used cross‐sectional baseline data from a household survey conducted in December 2017 as part of a maternal nutrition programme evaluation study (http://ClinicalTrials.gov Identifier: NCT03378141). The survey was carried out in two districts (Unnao & Kanpur Dehat) and 26 rural blocks, including 1,838 recently delivered women (RDW) with infants under 6 months of age. The sample was selected following a two‐stage cluster sampling technique that as follows: (a) selection of seven Gram Panchayats per block using probability proportional to size and (b) selection of up to 13 RDWs using systemic random sampling from a frame of 300–350 listed households. Within the household, 1,194 husbands and 1,353 MMILs of RDWs were also interviewed as part of the survey. The sample size for husbands and MMILs is lower than the RDW sample because husbands migrated or were unavailable due to working hours (*n* = 644), and MMILs did not live in the same household (*n* = 485). In 893 families, we have data on all three household members. Data from RDW were used to assess maternal, health service, and community factors related to breastfeeding practices, and data from husbands/MMILs were used to assess the role of family members to support women for breastfeeding practices.

### 2.2 Outcomes

Breastfeeding practices were assessed by using the following three indicators recommended by the WHO (2008): (a) EIBF, defined as the proportion of infants who were reported by mothers to have been put to the breast within 1 hr of birth; (b) prelacteal feeding, defined as the proportion of infants who were fed any foods or liquids other than breastmilk during the first 3 days after birth; and (c) EBF, defined as the proportion of infants 0–5.9 months of age who were fed only breast milk in the previous day (no foods, no liquids, with the exception of medications such as drops and syrups).

### 2.3 Independent variables

The selection of potential determinants of breastfeeding practices was considered at multiple levels, including maternal characteristics, health services access, and family/community support factors. We controlled for child age, child sex, maternal age, socio‐economic status, food insecurity, religion, and caste.

#### 2.3.1 Maternal characteristics

We include maternal behavioural determinants, maternal capabilities such as mental stress, decision‐making power, domestic violence, and postpartum symptoms as well as maternal demographic characteristics in the analyses.

Maternal determinants of breastfeeding practices were assessed though maternal knowledge and beliefs and self‐efficacy. Knowledge of EIBF was assessed based on mother's answers to questions related to early breastfeeding, such as when a baby should begin breastfeeding, benefits of early initiation, and use and benefits of colostrum. For knowledge of EBF, several topics were included: benefits and duration of EBF, specific practices to follow for EBF, breastfeeding in different conditions of mother's health, and duration to continue breastfeeding alongside complementary foods (Supplemental Table 1). Each knowledge item was given a score of 1 (correct) or 0 (incorrect), and the sums were used to construct a composite knowledge scores. For ease of interpretation, these scores were then divided into tertiles (as low‐, medium‐, or high‐knowledge levels) for multivariable analyses. Belief and self‐efficacy were measured on a 5‐point Likert scale by asking women the extent to which they agreed or disagreed with statements related to adopting recommended practices for breastfeeding (Nguyen et al., 2016). A scale representing maternal beliefs and self‐efficacy favouring EIBF or EBF was created for examination in the multivariable models (Supplemental Table 2).

Maternal mental distress was measured by the 20‐item Self Reporting Questionnaire which was scored 0 or 1 depending on responses related to perceived stresses in the last 30 days; a cut‐off of seven was used to categorise stress levels into high and low (Nguyen et al., 2014; WHO, 1994). Decision‐making power was measured based on mothers' responses to 16 questions related to women's roles in making decisions either alone or in conjunction with their husbands on purchasing food items, practices during pregnancy and for child care, and seeking health services (Supplemental Table 3). Each item was given a score of 1 or 0 and the sum of scores was divided to obtain high, medium, and low decision‐making categories. Domestic violence was assessed based on women's self‐reported experience of any violence as well as emotional, physical, or sexual violence during the last 12 months (Garcia‐Moreno, Jansen, Ellsberg, Heise, & Watts, 2005). Child birth weight, estimated based on maternal estimation and recall of child size at birth (i.e., smaller than average), was used as a proxy for low‐birth weight. Maternal and child weight (using a calibrated electronic scale) and height (using a portable stadiometer) were recorded at time of interview. Child height‐for‐age Z‐scores, weight‐for height, and weight‐for‐age were calculated according to 2006 WHO child growth standards (WHO, 2006). Maternal body mass index (kg/m^2^) was calculated and categorised as underweight (<18.5 kg/m^2^).

#### 2.3.2 Health service factors

Women were asked about whether they had an institutional delivery and mode of delivery [vaginal or caesarean section (C‐section)]. Breastfeeding counselling was assessed by asking women about various messages they received from front line workers during pregnancy and postpartum. Breastfeeding support was measured by asking women about the support they received at delivery in initiating breastfeeding.

#### 2.3.3 Family factors

Support from family members was assessed by asking husbands/MMILs of RDW about actions they took to support their wives/daughters for breastfeeding after delivery (Supplemental Table 4). Each question was given a score of 1 or 0 and the sum of scores was divided to obtain high‐, medium‐, and low‐support categories. We also measured husbands/MMILs knowledge on EIBF and EBF (using similar questions as for mothers) and categorised into tertiles as low‐, medium‐, and high‐knowledge levels.

#### 2.3.4 Community factors

Perceived social norms were measured on a 5‐point Likert scale by asking women the extent to which they agreed or disagreed about what other people in the community expected or thought a mother should do related to breastfeeding recommendations. The scales for social norms were constructed from five items by averaging responses to all items for each category, then divided into tertiles to obtain high‐, medium‐, and low‐norm categories.

### 2.4 Control variables

Additionally**,** we controlled for maternal age, religion (Hindu or others), caste (scheduled caste/schedule tribe or others), and education (categorised as illiterate, elementary, middle, and high school or higher), and child age and sex, and household socio‐economic status (SES). The SES variable was constructed using a principal components analysis of variables on housing conditions and asset holdings; the first component derived from component scores was used to divide the SES score into tertiles (Filmer & Pritchett, 2001; Vyas & Kumaranayake, 2006). Household food security index was calculated using the Household Food Insecurity Access Scale 9 item scale on household's experience of food insecurity in the past 30 days and coded as food secure versus insecure (Coates, Swindale, & Bilinsky, 2007).

### 2.5 Statistical analysis

Descriptive analysis was used to describe the characteristics of the study population. Bivariate analyses were conducted to test for associations between potential determinants with breastfeeding practices. Multivariable logistic regression models were applied to examine the association between the significant various determinants (at maternal, household, community, and health service levels) and outcomes, adjusting for control variables and accounting for variation among Gram Panchayats as a random effect using a cluster sandwich estimator. There was no evidence of multicollinearity in the models. Finally, population attributable risk analysis (Newson, 2013) was used to estimate by how much the key breastfeeding outcomes (EIBF, prelacteal feeding, and EBF) can be improved if select modifiable factors are changed based on regression models results under different scenarios (changing each determinant alone or the combination of determinants). All analysis was done using Stata version 15. Statistical significance was defined as *p* < .05.

## 3 RESULTS

As per the design of the study, all women had an infant between the ages of 0–5.9 months of age with an average age of 3.2 ± 1.5 months (Table 1). About a third of the women had completed high school, whereas 28% had no formal schooling. In contrast, 83% of MMILs and 18% of husbands had no formal schooling. The majority (80%) of women delivered in a health facility, and 11% women gave birth via a C‐section. The prevalence of reported low‐birth weight was 17%. More than a quarter (27%) of households experienced food insecurity at the time of the survey. Domestic violence in the last 12 months was high, where more than a third of women (36%) reported some form of violence (physical violence 27%, emotional violence 26%, and sexual violence 9%; Table 1).

**Table 1:** Maternal, household and community characteristics of study participants in Uttar Pradesh

Characteristics	Mean ± SD or percent
**Maternal characteristics**	
Maternal age	25.8 ± 4.3
Religion (Hindu)	93.3
Caste category	
Scheduled caste/tribe	41.0
Other backward classes	44.1
Others	14.9
Education	
No schooling	28.5
Elementary school	14.6
Middle school	22.3
≥ High school	34.6
Parity	2.24 ± 1.3
Thin mom (BMI < 18.5)	20.9
Mental stress score	3.05 ± 3.7
High mental stress >7	17.6
Knowledge score^a^	
Early initiation of breastfeeding	4.9 ± 2.0
Exclusive breastfeeding	3.3 ± 1.2
Overall breastfeeding	3.6 ± 1.2
Belief and self‐efficacy score^a^	7.5 ± 1.4
Decision making power score^a^	4.8 ± 3.4
Domestic violence experience (last 12 m)	
Physical violence	29.2
Sexual violence (ever)	9.5
Any violence	35.6
**Health services received**	
Delivery in health facility	80.3
C‐section	11.3
Received BF counselling during pregnancy	39.2
Received BF support at delivery	47.7
Received BF counselling during postpartum	21.3
**Family and community factors**	
Husband's education	
No schooling	17.8
Elementary school	12.7
Middle school	23.6
≥ High school	45.9
Husband's knowledge score^a^	
Early initiation of breastfeeding	3.9 ± 2.5
Exclusive breastfeeding	3.1 ± 1.3
Overall breastfeeding	3.2 ± 1.4
Husband's supports for BF score^a^	0.1 ± 0.5
Mothers/MIL's education	
No schooling	82.8
Elementary school	10.3
Middle school	3.8
≥ High school	3.2
Mothers/MIL's knowledge score^a^	
Early initiation of breastfeeding	4.1 ± 2.3
Exclusive breastfeeding	3.0 ± 1.2
Overall breastfeeding	3.2 ± 1.2
Mothers/MIL's supports for BF score^a^	0.8 ± 1.4
Social norm scores^1^	7.1 ± 1.5
**Household characteristics**	
Food insecurity	27.1
**Child characteristics**	
Age (months)	3.2 ± 1.5
Sex	
Male	51.3
Female	48.7
Low birth weight	17.3
HAZ	‐1.2 ± 1.5
WAZ	‐1.4 ± 1.3
WHZ	‐0.5 ± 1.6

*Note*. Sample size include 1,838 recently delivered women, 1,194 husbands and 1,353 MMIL.

Abbreviations: BF, breastfeeding; BMI, body mass index; C‐section, caesarean section; FLW, front line worker; HAZ, height‐for‐age Z‐score; MIL; mother‐in‐law; WAZ, weight‐for‐age Z‐score; WHZ, weight‐for‐height Z‐score.

a Scores were scaled and ranged from 0–10.

**Figure 1:**
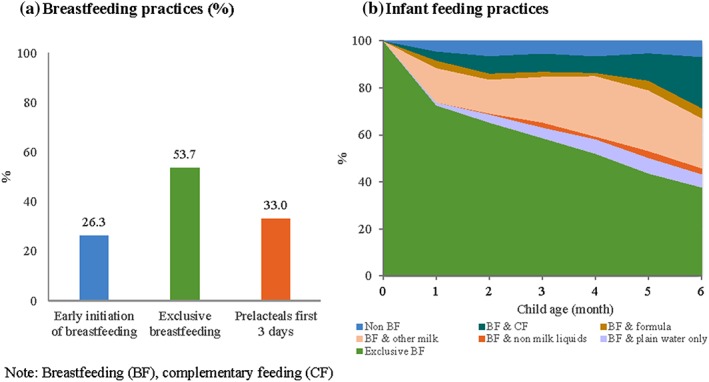
Prevalence of early initiation of breastfeeding, prelacteal feeding, and exclusive breastfeeding

Breastfeeding counselling was received by 39% of women during pregnancy and 21% of women postpartum. In addition, 47% of women reported receiving breastfeeding support at the time of delivery, and 75% of women were visited by a front line worker during the postpartum period. EIBF was reported by 26% of the women, and nearly a third reported their infant received a prelacteal fed to the infant in the first 3 days of life (Figure 1a). The most common prelacteal foods were cow/goat milk, honey and infant formula (results not shown). EBF was reported by about half of mothers, with a sharp decline with increasing child age (Figure 1b). The most common substitute for mother's breastmilk was other animal milk (21.0%), followed by inappropriate early introduction of complementary foods (10.1%), water (5.0%), formula (2.8%), and nonmilk liquids (1.6%).

The key maternal, health service, family, and community factors associated with early breastfeeding practices are presented in Tables 2, 3, 4. For EIBF, maternal knowledge was a critical factor, with medium and high knowledge being associated with a three‐ to four‐fold increase in EIBF compared with low knowledge (Table 2). Additionally, high maternal beliefs and self‐efficacy, high MMIL knowledge, and high parity (≥3) were positively associated with EIBF; these associations were attenuated in the adjusted models. Mothers who gave birth in a health facility were more likely to report EIBF [odds ratio (OR): 95% confidence interval (CI); 1.68: 1.17–2.42], whereas women who had a C‐section were 68% less likely to (OR: 95% CI; 0.32: 0.18–0.55). Mothers who received breastfeeding counselling during pregnancy and received support at delivery were ~1.4 times more likely to initiate breastfeeding in the first hour. Similar results were found when analysis was restricted to only normal deliveries.

**Table 2:** Maternal, household, community, and health service factors associated with early initiation of breastfeeding

	Bivariate analysis (*N* = 1838)			Multivariable analysis			
				Maternal and health factors (*n* = 1,837)	All factors (*n* = 1,353)		
	OR	95% CI	*P* value	OR	95% CI	OR	95% CI
**Maternal characteristics**							
Knowledge of EIBF (ref = low)			.0001				
Medium	3.48***	2.50, 4.85		3.40***	2.39, 4.84	3.05***	2.03, 4.58
High	4.95***	3.54, 6.90		4.85***	3.41, 6.89	4.17***	2.71, 6.40
Beliefs and self‐efficacy for BF			.0062				
Medium	1.24	0.98, 1.57		1.08	0.83, 1.40	1.03	0.75, 1.39
High	1.44*	1.02, 2.04		1.26	0.84, 1.88	1.20	0.74, 1.93
Maternal education (ref = no schooling)			.0221				
Elementary school	1.19	0.87, 1.64					
Middle school	0.88	0.66, 1.16					
≥ High school	0.92	0.71, 1.19					
Parity (ref = 1)			.0096				
2	1.02	0.78, 1.35		0.93	0.68, 1.27	1.03	0.73, 1.46
≥3	1.34**	1.07, 1.69		1.28	0.89, 1.84	1.43	0.91, 2.22
Low BMI	1.12	0.89, 1.43	.3190				
Mental stress (high)	0.83	0.62, 1.10	.1980				
Domestic violence (last 12 months)	1.14	0.91, 1.42	.2510				
**Health service factors**							
Institutional delivery	1.99***	1.51, 2.61	.0001	1.96***	1.46, 2.63	1.68**	1.17, 2.42
C‐section	0.28***	0.17, 0.47	.0001	0.25***	0.15, 0.42	0.32***	0.18, 0.55
Breastfeeding counselling pregnancy	1.80***	1.43, 2.26	.0001	1.40*	1.08, 1.81	1.38*	1.03, 1.85
Breastfeeding support at delivery	1.78***	1.42, 2.24	.0001	1.40**	1.09, 1.79	1.43*	1.05, 1.93
**Family and community factors**							
Husband's education			.0547				
Elementary school	1.44	0.86, 2.42					
Middle school	1.42	0.93, 2.16					
≥ High school	1.24	0.85, 1.82					
Husband's knowledge of EIBF (ref = low)			.0539				
Medium	1.01	0.73, 1.39					
High	1.17	0.86, 1.61					
MMIL's education			.1419				
Elementary school	0.85	0.54, 1.32					
Middle school	1.27	0.67, 2.41					
≥ High school	1.22	0.60, 2.46					
MMIL's knowledge of EIBF			.0003				
Medium	1.01	0.76, 1.35				0.83	0.60, 1.14
High	1.88***	1.41, 2.51				1.32	0.95, 1.82
Social norms (ref = low)			.0252				
Average	0.90	0.63, 1.30					
High	0.96	0.65, 1.41					

*Note*. Model is adjusted for religion, caste, child age, child sex, maternal age, socio‐economic status, and food insecurity.

Abbreviations: BF, breastfeeding; BMI, body mass index; C‐section, caesarean section; CI, confidence interval; EIBF, early initiation of breastfeeding; MMIL, mother/mother‐in‐law; OR, odds ratio.

* *p* < .05.

** *p* < .01.

*** *p* < .001.

**Table 3:** Maternal, household, community, and health service factors associated with prelacteal feeding

	Bivariate analysis (*N* = 1838)			Multivariable analysis			
				Maternal and health factors (*n* = 1837)		All factors (*n* = 870)	
	OR	95% CI	*P* value	OR	95% CI	OR	95% CI
**Maternal capacity**							
Knowledge of EBF (ref = low)			.0001				
Medium	0.61***	0.48, 0.76		0.64**	0.49, 0.84	0.44***	0.29, 0.67
High	0.35***	0.27, 0.45		0.35***	0.26, 0.47	0.29***	0.19, 0.45
Belief and self‐efficacy for BF			.0001				
Medium	0.81	0.65, 1.02		0.96	0.75, 1.22	1.30	0.90, 1.87
High	0.47***	0.33, 0.68		0.58**	0.41, 0.84	0.45**	0.24, 0.82
Maternal education (ref = no schooling)			.0173				
Elementary school	1.03	0.76, 1.40					
Middle school	0.97	0.75, 1.25					
≥ High school	0.79	0.61, 1.02					
Parity (ref = 1)			.0001				
2	0.71**	0.55, 0.91		0.70*	0.53, 0.92	0.96	0.61, 1.51
≥3	0.77*	0.61, 0.97		0.64**	0.46, 0.89	0.98	0.56, 1.72
Low BMI	1.11	0.87, 1.42	.3890				
Mental stress (high)	1.05	0.81, 1.36	.7240				
Domestic violence (last 12 months)	1.08	0.88, 1.34	.4610				
**Health service factors**							
Institutional delivery	0.38***	0.30, 0.48	.0001	0.35***	0.26, 0.46	0.31***	0.20, 0.47
C‐section	2.72***	2.08, 3.56	.0001	3.39***	2.49, 4.60	3.84***	2.40, 6.15
Breastfeeding counselling pregnancy	0.61***	0.49, 0.76	.0001	0.76*	0.60, 0.97	0.77	0.52, 1.14
Breastfeeding support at delivery	0.48***	0.39, 0.59	.0001	0.60***	0.48, 0.76	0.70	0.48, 1.01
Breastfeeding counselling postpartum	0.72**	0.55, 0.94	.0001	1.00	0.74, 1.34	0.92	0.57, 1.48
**Family and community factors**							
Husband's education			.2760				
Elementary school	1.14	0.71, 1.84					
Middle school	0.88	0.59, 1.31					
≥ High school	0.86	0.60, 1.24					
Husband's knowledge of EBF			.0001				
Medium	0.74*	0.56, 0.97				0.87	0.59, 1.27
High	0.70*	0.52, 0.95				1.03	0.65, 1.62
MMIL's education			.0027				
Elementary school	0.62*	0.41, 0.92				0.53*	0.30, 0.96
Middle school	0.99	0.52, 1.90				0.86	0.35, 2.09
≥ High school	0.63	0.29, 1.37				0.46	0.14, 1.52
MMIL's knowledge of EBF			.0003				
Medium	0.81	0.62, 1.05				1.28	0.88, 1.86
High	0.64**	0.47, 0.85				1.07	0.70, 1.62
Social norms (ref = low)			.0001				
Average	0.79	0.58, 1.07				0.74	0.47, 1.18
High	0.43***	0.30, 0.62				0.57	0.32, 1.02

*Note*. Model is adjusted for religion, caste, child age, child sex, maternal age, socio‐economic status, and food insecurity.

Abbreviations: BF, breastfeeding; BMI, body mass index; C‐section, caesarean section; CI, confidence interval; EIBF, early initiation of breastfeeding; MMIL, mother/mother‐in‐law; OR, odds ratio.

* *p* < .05.

** *p* < .01.

*** *p* < .001.

**Table 4:** Maternal, household, community and health service factors associated with exclusive breastfeeding

	Bivariate analysis (*N* = 1838)			Multivariable analysis			
				Maternal and health factors (*n* = 1837)		**All factors** **(*n* = 870)**	
	OR	95% CI	*P* value	OR	95% CI	OR	95% CI
**Maternal capacity**							
Knowledge on EBF (ref = low)			.0001				
Medium	1.74***	1.37, 2.21		1.63***	1.26, 2.11	1.46*	1.07, 2.00
High	2.18***	1.69, 2.80		2.10***	1.60, 2.77	1.72**	1.22, 2.43
Belief and self‐efficacy for BF			.0018				
Medium	1.35**	1.10, 1.66		1.29*	1.03, 1.61	1.17	0.90, 1.54
High	2.09***	1.52, 2.88		1.93***	1.37, 2.71	2.09***	1.35, 3.24
Maternal education (ref = no schooling)			.6003				
Elementary school	0.80	0.58, 1.12					
Middle school	0.87	0.65, 1.16					
≥ High school	0.90	0.71, 1.16					
Parity (ref = 1)			.0850				
2	1.26*	1.00, 1.60		1.42**	1.10, 1.82	1.25	0.89, 1.73
≥3	1.27*	1.02, 1.59		1.86***	1.39, 2.50	1.73**	1.16, 2.58
Low BMI	0.89	0.72, 1.10	.4660				
Mental stress (high)	0.58***	0.45, 0.76	.0001	0.61***	0.45, 0.81	0.62**	0.43, 0.89
Domestic violence (last 12 months)	0.73**	0.60, 0.90	.0020	0.79*	0.63, 0.99	0.75*	0.57, 0.99
**Health service factors**							
Institutional delivery	1.03	0.82, 1.30	.6332				
C‐section	0.62***	0.47, 0.82	.0010	0.65**	0.48, 0.88	0.61*	0.40, 0.92
Breastfeeding counselling pregnancy	0.82	0.67, 1.01	.2334				
Breastfeeding support at delivery	1.05	0.86, 1.29	.6218				
Breastfeeding counselling postpartum	0.87	0.75, 1.02	.1263				
**Family and community factors**							
Husband's education			.4043				
Elementary school	0.94	0.60, 1.47					
Middle school	0.86	0.60, 1.25					
≥ High school	0.92	0.67, 1.27					
Husband's knowledge of EBF			.0940				
Medium	1.23	0.95, 1.60				1.08	0.82, 1.42
High	1.34*	1.03, 1.75				1.27	0.94, 1.71
MMIL's education			.2279				
Elementary school	0.76	0.53, 1.09					
Middle school	0.78	0.21, 1.50					
≥ High school	0.65	0.33, 1.27					
MMIL's knowledge of EBF			.1841				
Medium	1.07	0.80, 1.42					
High	0.83	0.65, 1.07					
Social norms (ref = low)			.0544				
Average	1.21	0.85, 1.71				1.19	0.76, 1.87
High	1.69**	1.16, 2.46				1.34	0.81, 2.22

*Note*. Model is adjusted for religion, caste, child age, child sex, maternal age, socio‐economic status, and food insecurity.

Abbreviations: BF, breastfeeding; BMI, body mass index; C‐section, caesarean section; CI, confidence interval; EIBF, early initiation of breastfeeding; MMIL, mother/mother‐in‐law; OR, odds ratio.

* *p* < .05.

** *p* < .01.

*** *p* < .001.

Key factors associated with an infant receiving a prelacteal feed in first 3 days is presented in Table 3. Maternal knowledge of EBF significantly reduced the odds of prelacteal feeding by 56% (OR: 95% CI; 0.44: 0.29–0.67) and 71% (OR: 95% CI; 0.29: 0.19–0.45) among women with medium and high levels of knowledge, respectively compared with low knowledge. Likewise, women with high beliefs and self‐efficacy for breastfeeding were 55% less likely to provide a prelacteal feed to their infant (OR: 95% CI; 0.45: 0.24–0.82). Delivering in health facility significantly decreased the odds of prelacteal feeding (OR: 95% CI; 0.31: 0.20–0.47), whereas having a C‐section increased the odds (OR: 95% CI; 3.84: 2.40–6.15). Breastfeeding counselling during pregnancy and support during delivery were significant in the bivariate and the maternal and health factor model (OR: 95% CI; 0.76: 0.60–0.97 and OR: 95% CI; 0.60: 0.48–0.76, respectively) but not in full model with family and community factors, with the restricted sample size. Women whose MMILs had an elementary school education compared with no education were less likely to provide a prelacteal feed to their infant (OR: 95% CI; 0.53: 0.30–0.96). In the bivariate models, there was some indication that husbands and MMIL knowledge of EBF as well as high social norms may be protective against prelacteal feeding, although these factors were not significant in full model. Similar results were found when analysis was restricted to only normal deliveries.

Key factors associated with EBF are described in Table 4. Again, we noted a stepwise progression of higher maternal knowledge increasing the odds of EBF in the first 6 months of life (OR: 95% CI; 1.46: 1.07–2.00 and OR: 95% CI; 1.72: 1.22–2.43, for medium and high knowledge, respectively, compared with low knowledge). Likewise, women with high maternal beliefs and self‐efficacy were twice more likely to EBF (OR: 95% CI; 2.09: 1.35–3.24). Women with a higher parity (≥3 compared with 1) were also more likely to EBF their infant (OR: 95% CI; 1.73: 1.16–2.58). However, women with high levels of stress were 38% less likely to EBF (OR: 95% CI; 0.62: 0.43–0.89). Women who experienced domestic violence in the last 12 months were also 25% less likely to EBF their infant (OR: 95% CI; 0.75: 0.57–0.99). Furthermore, women of higher SES were 50% more likely to provide breastmilk substitutes to their infant (OR: 95% CI; 0.50: 0.36–0.69 for high vs. low SES). Women who delivered via C‐section were 40% less likely to EBF (OR: 95% CI; 0.61: 0.40–0.92). In the bivariate models, there was some indication that husband's knowledge of EBF as well as high social norms may help promote EBF, although these factors were not significant in full model. MMIL education and knowledge was not significantly associated with EBF.

**Figure 2:**
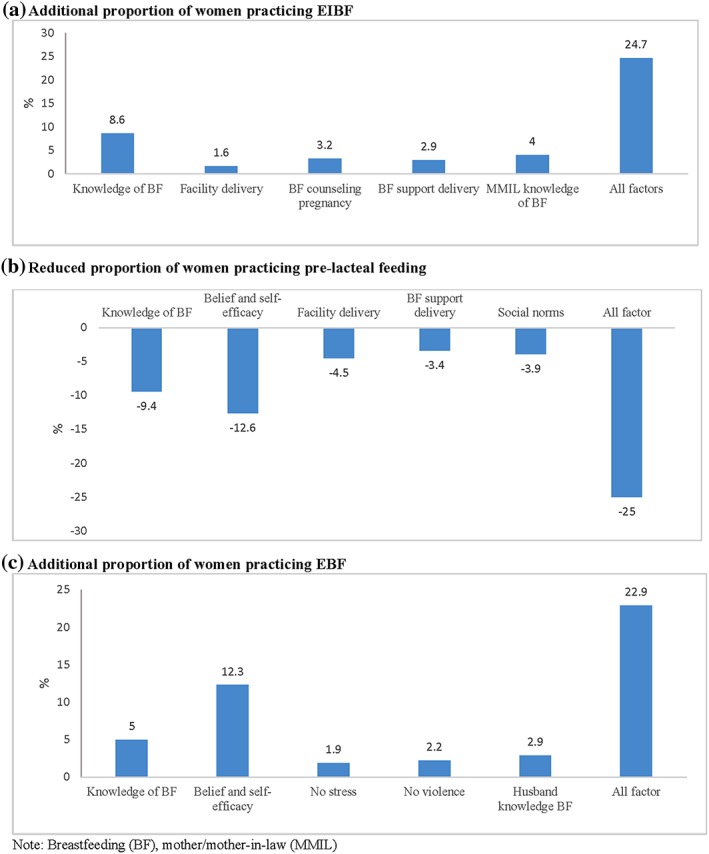
Population attributable risk estimations of the influence of select modifiable factors on breastfeeding practices

We modelled the population attributable risk estimation for the influence of significant modifiable factors on breastfeeding practices (Figure 2). This allows us to understand potential influence of programme on improving breastfeeding in this context. We estimate that EIBF can be improved by additional 25 percentage points (pp) by improving mother and MMIL breastfeeding knowledge, increasing facility deliveries, and providing breastfeeding counselling during pregnancy and support at delivery. Prelacteal feeding could potentially be reduced by 25pp improving mother's breastfeeding knowledge, beliefs and self‐efficacy, increasing facility delivery, providing breastfeeding support at delivery, and positively influencing social norms in community. In addition, we estimate that EBF rates can be increased by 23pp through increasing maternal and husband breastfeeding knowledge, reducing maternal stress and domestic violence, and enhancing maternal breastfeeding beliefs and self‐efficacy.

## 4 DISCUSSION

In our study, less than one third of infants were breastfed within the first hour of birth and one third were receiving a prelacteal feed. In addition, only half of the women were reaching the goal of EBF in the first 6 months of life. Although these poor early breastfeeding practices are concerning, our study identifies several key determinants that influence breastfeeding practices in Uttar Pradesh, India, many of which are modifiable. EIBF was positively associated with maternal knowledge, counselling during pregnancy/delivery, and vaginal delivery at a health facility. Prelacteal feeds were less likely to be given to infants when the mothers had higher knowledge, beliefs and self‐efficacy, and delivered at a health facility, and MMILs had attended school. EBF was positively associated with maternal knowledge, beliefs and self‐efficacy, parity and socio‐economic status. High maternal stress and domestic violence contributed to lower EBF. We estimate that with improving maternal and family knowledge of breastfeeding, improving counselling on breastfeeding and providing support at delivery, improving self‐efficacy, reducing maternal stress and domestic violence can together improve EIBF by 25pp, prelacteal feeding can be reduced by 25pp, and EBF can be increased by 23pp.

A key strength of this study was a focus on the multilevel determinants including: maternal, health service, family, and community level factors associated with early feeding practices. This is in alignment with the Lancet breastfeeding series that outlines the complex factors that are required to best support women and create an enabling environment at the societal level, within the health system, in the community/workplace, and within families (Rollins et al., 2016). Previous studies from India on factors influencing breastfeeding practices (Gayhane et al., 2018; Sandor & Dalal, 2013) have had similar findings. Our study builds upon this work by collecting firsthand information from husbands and MMILs directly in addition to mothers.

### 4.1 Maternal and household factors

In our study, high maternal breastfeeding knowledge was positively associated with all three outcomes of early breastfeeding practices (over 4 times more likely to EIBF, nearly twice as likely to EBF, and 71% less likely to practice prelacteal feeding, compared with women with low knowledge). Women with high maternal beliefs and self‐efficacy were 2 times more likely EBF and was associated with a 55% reduction in prelacteal feedings. This is consistent with prior research in other contexts, where mothers with greater breastfeeding knowledge and ability to make informed decisions had improved breastfeeding practices (Egata, Berhane, & Worku, 2013; Maonga, Mahande, Damian, & Msuya, 2016; Mogre, Dery, & Gaa, 2016). Likewise, in a systematic review, maternal breastfeeding self‐efficacy was identified as a key modifiable factor directly associated with increased odds of EBF (Brockway, Benzies, & Hayden, 2017). In the current study, maternal education was not associated with any of the breastfeeding outcomes. In contrast, a study of in‐hospital deliveries in India found that higher maternal education was associated with a two‐fold increase in EIBF and decreased odds of prelacteal feeding (Patel, Banerjee, & Kaletwad, 2013). In an analysis of India's NFHS‐3 2005–2006, higher maternal education associated with increased odds of EIBF but lower odds of EBF. Similarly, in a systematic review of the determinants of EIBF women across South Asia (Bangladesh, India, Nepal, and Pakistan) with no formal education were more likely to have delayed initiation of breastfeeding (Sharma & Byrne, 2016).

We found that higher SES was associated with a 50% decrease in EBF. Likewise, in a recent analysis of India's NFHS 2005–2006 and NFHS 2015–2016 datasets higher SES was negatively associated with EBF both directly and indirectly through access to information and services, parity, and urban/rural residence (Nguyen et al., 2018). The systematic review of determinants of EIBF in South Asia highlights differences in the role of sociodemographic factors by region and thus the need for context‐specific understanding on breastfeeding determinants (Sharma & Byrne, 2016). For example, delayed breastfeeding initiation was associated with lower SES in Bangladesh, but higher SES in Sri Lanka (Mihrshahi et al., 2010; Seranath et al., 2012).

Women who experienced domestic violence or high levels of stress were 25% to 38% less likely to exclusively breastfeed. In a systematic review of studies from different countries, including two from India, 8 of the 12 studies reviewed reported intimate partner violence to be associated with low breastfeeding intention, initiation, and EBF (Mezzavilla, Ferreira, Curioni, Lindsay, & Hasselmann, 2018). In India, women who were exposed to any intimate partner violence and to any physical or sexual violence were 22–26% less likely to exclusively breastfeed their infant (Zureick‐Brown, Lavilla, & Yount, 2015). These findings are concerning, given over a third of women in our population reported experiencing domestic violence in the past year, confirming statewide estimates for Uttar Pradesh, where 37% of women reported experiencing spousal violence (IIPS, 2017b). In a recent study in Bangladesh, breastfeeding counselling was shown to mitigate some of the negative affect of domestic violence on breastfeeding outcomes and provide support for vulnerable women (Frith et al., 2017). Although counselling may be beneficial, there is urgent need to tackle the larger issue and address violence against women in India.

### 4.2 Health services factors

Breastfeeding counselling during pregnancy and support at delivery were positively associated with EIBF (OR 1.38, 1.43, respectively). For prelacteal feeds, counselling during pregnancy and support at delivery was significant in bivariate and adjusted multivariable models with maternal health factors but not in the full model including family and community factors, although trend remains. However, EBF was not associated with breastfeeding counselling and support. Although EIBF is accomplished by feeding a baby within the first hour after delivery, EBF is consistent breastfeeding for 6 months and hence the challenges associated with these two are connected and yet distinct practices are potentially different. Providing support right after birth to facilitate EIBF and counselling postpartum are therefore able to promote EIBF. Counselling and support at birth and postpartum are one‐time activities, which are unlikely to help mothers with challenges throughout the 6 months, which can originate at the individual, family, or community levels or a combination of all. This suggests the need for an intense and continued support for ensuring EBF.

Consistent with prior literature (Adhikari, Khanal, Karkee, & Gavidia, 2014; Roy, Mohan, Singh, Singh, & Srivastava, 2014), our findings showed that women who delivered in a health facility were 1.7 times more likely to EIBF and had a 69% reduction in prelacteal feedings (69%) compared with those who gave birth at home. These are promising trends given government efforts to increases institution deliveries (currently at 80% among women in study).

Women who had a C‐section were at greater risk for poor breastfeeding outcomes compared with women who had a vaginal delivery. Women with C‐section had 68% reduction in EIBF, 40% reduction in EBF, and were 3.8 times more likely to provide infant with a prelacteal feed. This is consistent with findings from a large secondary analysis of the WHO Global Survey in 24 countries where infants delivered by C‐sections where 72% less likely to be breastfeed within the first hour of birth (Takahashi et al., 2017). Likewise, in an analysis of in‐hospital deliveries in India, women who delivered via C‐section were 2.5 times more likely to provide their infant with a prelacteal feed (Patel et al., 2013). It is critical to address the emerging concern of C‐section and risk of poor breastfeeding outcomes in India, through both emphasising the importance that C‐sections are only done when medically indicated, as well as having clear operational guidance on breastfeeding support among C‐section deliveries.

### 4.3 Family and community factors

The role of MMILs, husbands, and community norms on breastfeeding practices was critical in our bivariate models, but the associations were attenuated in the multivariable models. This may be due to the smaller sample size in the full model or potentially some of these factors operate through other variables in the model and merits closer examination in future work. Prior research has identified both grandmothers and fathers as being influential on breastfeeding practices (Negin et al., 2016; Arora, McJunkin, Wehrer, & Kuhn, 2000; Bromberg & Darby 1997; Pisacane, Continisio, Aldinucci, D'Amora, & Continisio, 2005). We also see a positive influence of high community social norms with reduced odds of providing prelacteal feed and increased odds of EBF in bivariate models. Despite modest results, purposefully engaging family and community members is critical to improving breastfeeding practices. As highlighted the Global Breastfeeding Collective lead by UNICEF and WHO, “*Breastfeeding isn't just a one women job*.” (UNICEF, 2017), breastfeeding is a collective responsibility of family and communities, health care providers, and skilled lactation counsellors that need to work together to address the social, political, and environmental barriers to breastfeeding.

Although this study provides a robust multifactorial approach to both understanding key determinants of early breastfeeding practices and modelling potential programme impact, there are some important limitations. Our sample size was reduced in models that included family and community factors, which may have reduced our power and ability to detect significant differences in full models. To address this limitation, we provided three models, bivariate, maternal, and health factors and all factors to allow for comparisons. Characteristics of women in the full and reduced sample were similar. Our indicators for support do not capture other indirect aspects of support including helping with older children, housework and so forth that may allow the mother to breastfeed the baby. Future research should include facility‐level information on hospital adherence to baby‐friendly practices to better understand variation in breastfeeding support for women. Although progress is being made in the scale‐up of the baby‐friendly hospital initiation in India; limited data suggest low implementation in Uttar Pradesh (Gupta, 2000). Additional qualitative data may allow for more in‐depth understanding of women's perceptions and influential factors surrounding breastfeeding (Sharma, 2016).

## 5 CONCLUSION

Our study provides insights into the multiple factors that influence EIBF, prelacteal feeding, and EBF in Uttar Pradesh, and re‐emphasise that supporting breastfeeding truly takes a village. Many of the factors identified are modifiable and will be directly targeted as part of the larger state and national programmes in India designed to create an enabling environment for better breastfeeding outcomes (Sahu, 2018; Kapil, 2002; Lim et al., 2010; UNICEF, 2016; NITI Aayog, 2017). However, there is a history of strong policies and programmes but weak programme implementation and low adherence to recommendations (Avula et al., 2017). A focus on strong programme implementation to address the key barriers and facilitators identified will aid in accelerating previous gains and help reach breastfeeding goals in Uttar Pradesh.

## FUNDING

The Bill & Melinda Gates Foundation, through Alive & Thrive, managed by FHI 360.

## CONFLICT OF INTEREST

The authors declare that they have no conflicts of interest.

## CONTRIBUTOR STATEMENT

All authors read and approved the final draft.

## Supporting information

Table S1: Knowledge on early initiation of breastfeeding and exclusive breastfeeding

Table S2: Belief, self‐efficacy and social norms related to breastfeeding practices

Table S3: Decision making power

Table S4: Family support for breastfeeding
