# Supplementary material for: It takes a village: An empirical analysis of how husbands, mothers‐in‐law, health workers, and mothers influence breastfeeding practices in Uttar Pradesh, India
Source: Matern Child Nutr. 2019 Nov 26;16(2):e12892. doi: 10.1111/mcn.12892 (PMC7083414; doi:10.1111/mcn.12892)
Supplement: Supplementary file 1 — Table S1: Knowledge on early initiation of breastfeeding and exclusive breastfeeding [file MCN-16-e12892-s001.docx]

**Supplemental Table 1: Knowledge on early initiation of breastfeeding and exclusive breastfeeding**

| **Knowledge** | **RDW (n=1838)**  **%** | **Husband (n=1233)**  **%** | **MMIL (n=1467)**  **%** |
| --- | --- | --- | --- |
| **Early initiation of breastfeeding** |  |  |  |
| A baby start breastfeeding within an hour after birth | 75.95 | 59.12 | 62.24 |
| Reasons for starting BF early |  |  |  |
| Initiating breastfeeding within 1 hour can save baby’s life | 27.86 | 21.57 | 21.88 |
| Initiating breastfeeding within 1 hour can reduce mothers bleeding | 7.67 | 10.14 | 10.02 |
| Initiating breastfeeding within 1 hour can improve breastmilk supply | 4.46 | 6.89 | 6.95 |
| Colostrum is good for the baby | 29.49 | 23.76 | 24.47 |
| Initiating breastfeeding within 1 hour is good for the child’s health | 24.10 | 15.17 | 17.45 |
| Mother should feed baby colostrum soon after birth | 83.95 | 66.42 | 70.89 |
| Benefit of colostrum |  |  |  |
| Protects against allergies and infections | 28.78 | 25.47 | 21.40 |
| Provide good source of nutrition | 24.70 | 23.52 | 21.27 |
| Helps to prevent jaundice | 8.05 | 6.08 | 6.00 |
| Helps in passing of first stools or meconium | 1.74 | 3.16 | 3.54 |
| **Exclusive breastfeeding** |  |  |  |
| What can a baby under the age of 6 months be fed (breastmilk only) | 65.51 | 63.42 | 53.31 |
| How many months should a baby be breastfed exclusively (6 months) | 74.81 | 63.58 | 59.24 |
| Benefit of exclusive breastfeeding |  |  |  |
| Protects baby from illness | 49.35 | 44.77 | 39.54 |
| Helps baby grow and develop better | 27.69 | 26.60 | 25.09 |
| Provides a superior source of nutrients | 8.92 | 11.35 | 9.07 |
| Easy to digest | 8.60 | 9.25 | 9.88 |
| Provides adequate water for a baby in the six months | 4.03 | 6.24 | 5.52 |
| Clean, always ready and of a good temperature | 3.65 | 6.89 | 3.75 |
| Stimulates brain development of the baby | 7.56 | 10.06 | 6.95 |
| Delays a new pregnancy | 4.03 | 4.06 | 4.77 |
| Stimulates breast milk production | 5.50 | 5.43 | 5.93 |
| Saves money | 6.75 | 6.49 | 6.54 |
| Increased emotional bonding between mother and child | 5.60 | 4.14 | 5.04 |
| Good for mother’s health (decreases breast & ovarian cancer) | 5.60 | 3.89 | 4.09 |
| Empty one breast before switching to the other during breastfeeding | 6.96 | 6.89 | 6.07 |
| Breastfeed whenever the baby wants | 14.42 | 14.11 | 15.54 |
| A mother with small breasts can produce enough milk | 65.89 | 39.98 | 70.28 |
| A mother who is not well fed can produce enough milk | 21.27 | 13.46 | 19.84 |
| Actions if mother thinks her child is not getting enough breast milk |  |  |  |
| Breastfeed more frequently | 8.16 | 5.43 | 6.13 |
| Ensure proper position and attachment at the breast | 4.52 | 4.87 | 4.43 |
| Take enough time for baby to complete breastfeeding | 5.77 | 6.81 | 8.04 |
| Empty one breast before switching to the other | 3.70 | 6.08 | 6.20 |
| Mother needs to drink more water | 9.36 | 15.98 | 15.75 |
| Mother needs to eat more food | 32.54 | 38.36 | 39.06 |
| Mother needs to eat special foods | 25.35 | 19.63 | 24.40 |
| Mother needs to rest more | 4.79 | 3.89 | 2.79 |
| How to understand that child is getting sufficient milk |  |  |  |
| Baby urinates at least 6-7 times in a day | 6.09 | 6.73 | 7.02 |
| Child gaining weight and growing well | 7.83 | 14.27 | 12.34 |
| Infants (<6m) should not be given water even if weather is hot | 21.38 | 29.68 | 15.61 |
| Infants should not be given water to clean the mouth after breastfeeding | 36.18 | 41.44 | 27.88 |
| A mother should not stop breastfeeding if she becomes pregnant again | 31.77 | 44.93 | 29.11 |
| Give mother’s expressed breastmilk only if she is away | 1.52 | 2.19 | 1.43 |
| A mother should continue breastfeeding if she becomes ill | 30.79 | 27.49 | 24.68 |
| Begin complementary feeding after 6 months | 79.05 | 65.86 | 70.35 |
| Continue breastfeeding until child is 24 months | 51.58 | 43.63 | 53.31 |

* Each item was given a score of 1 (correct knowledge) or 0 (incorrect knowledge) and the sum of scores was divided to obtain high, medium, and low knowledge categories.
