# Supplementary material for: It takes a village: An empirical analysis of how husbands, mothers‐in‐law, health workers, and mothers influence breastfeeding practices in Uttar Pradesh, India
Source: Matern Child Nutr. 2019 Nov 26;16(2):e12892. doi: 10.1111/mcn.12892 (PMC7083414; doi:10.1111/mcn.12892)
Supplement: Supplementary file 2 — Table S2: Belief, self‐efficacy and social norms related to breastfeeding practices [file MCN-16-e12892-s002.docx]

**Supplemental Table 2: Belief, self-efficacy and social norms related to breastfeeding practices**

| **Items** | **%** |
| --- | --- |
| **Belief** |  |
| If I breastfeed my infant within 1 hour after giving birth, it’ll be good for my health and my child’s health | 86.8 |
| If I feed my infant a combination of breast milk and infant formula until s/he completes 6 months, I am giving him/her the **BEST** possible nutrition ^a^ | 53.5 |
| **Self-efficacy** |  |
| My body can produce enough breast milk to feed my newborn within one hour after birth. | 81.5 |
| My breast milk is of good enough quality to nourish my infant so that the infant does not need any other food, water, or infant formula until s/he has completed 6 months. | 73.8 |
| If my mother-in-law wants to feed my newborn infant formula in the first 24 hours after birth, I can refuse it | 61.7 |
| **Social norms** |  |
| Most people who are important to me (e.g. family members, friends…) think that a mother can breastfeed her infant within 1 hour after birth | 77.1 |
| Most people who are important to me (e.g. family members, friends…) think that I should feed my infant only breast milk, and no other food, water, or infant formula for the first 6 months | 70.5 |
| Most people who are important to me (e.g. family members, friends…) think that a baby should be given infant formula before she/he reaches 6 months of age^a^ | 42.9 |
| Most people who are important to me (e.g. family members, friends…) think that a baby should be given semi-solid food before she/he reaches 6 months of age^a^ | 38.4 |

^a^Reverse coded to create composite scores for beliefs/norms favoring BF.

* Each item was given a score of 1 or 0 and the sum of scores was divided to obtain high, medium, and low belief and self-efficacy categories.
