# Supplementary material for: It takes a village: An empirical analysis of how husbands, mothers‐in‐law, health workers, and mothers influence breastfeeding practices in Uttar Pradesh, India
Source: Matern Child Nutr. 2019 Nov 26;16(2):e12892. doi: 10.1111/mcn.12892 (PMC7083414; doi:10.1111/mcn.12892)
Supplement: Supplementary file 3 — Table S3: Decision making power [file MCN-16-e12892-s003.docx]

**Supplemental Table 3: Decision making power**

| **Items** | **Women**  **(n=1838)**  **%** |
| --- | --- |
| ***Women had decision, or women and her husband had joined decision on the following things:*** | |
| Buying of food like rice, vegetables | 31.45 |
| Buying animal source foods (meat, fish, poultry, eggs)? | 25.30 |
| Buying cooking oil | 32.10 |
| Buying medicine for yourself | 38.52 |
| Buying medicine for the children | 39.77 |
| What food is prepared every day? | 44.61 |
| If you have to work to earn money? | 29.82 |
| Visiting other family members, friends or relatives? | 32.26 |
| Seeing a doctor or visiting a dispensary when you are pregnant? | 42.22 |
| Use of family planning methods? | 61.92 |
| To eat nutritious food during pregnancy | 52.45 |
| To take supplemental tablets (IFA, Calcium) during pregnancy | 59.96 |
| To take rest every day for a certain time during pregnancy | 69.42 |
| Whether or not you breastfeed the child and when to give weaning food to the child? | 72.74 |
| What and how to feed the infant in his first year of life? | 70.78 |
| When to seek care for a sick child? | 68.66 |

*Each item was given a score of 1 or 0 and the sum of scores was divided to obtain high, medium, and low decision making categories.
