# Supplementary material for: It takes a village: An empirical analysis of how husbands, mothers‐in‐law, health workers, and mothers influence breastfeeding practices in Uttar Pradesh, India
Source: Matern Child Nutr. 2019 Nov 26;16(2):e12892. doi: 10.1111/mcn.12892 (PMC7083414; doi:10.1111/mcn.12892)
Supplement: Supplementary file 4 — Table S4: Family support for breastfeeding [file MCN-16-e12892-s004.docx]

**Supplemental Table 4: Family support for breastfeeding**

| **Items** | **Husband (n=1233)**  **%** | **MMIL**  **(n=1467)**  **%** |
| --- | --- | --- |
| Placed baby on mother’s chest immediately after delivery | 0.81 | 7.09 |
| Helped and told mother to breastfeed baby within 1 hour of birth | 1.70 | 8.66 |
| Did not give and advised not to give pre-lacteals (honey, water, cow or goat’s milk etc.) | 0.65 | 3.82 |
| Showed the right way of positioning and attaching the baby for breastfeeding | 0.32 | 7.09 |
| Helped to place the baby on the breast | 1.22 | 14.45 |

*Each item was given a score of 1 or 0 and the sum of scores was divided to obtain high, medium, and low support categories.
